# Ground far-red sun-induced chlorophyll fluorescence and vegetation indices in the US Midwestern agroecosystems

**DOI:** 10.1038/s41597-024-03004-w

**Published:** 2024-02-22

**Authors:** Genghong Wu, Kaiyu Guan, Hyungsuk Kimm, Guofang Miao, Xi Yang, Chongya Jiang

**Affiliations:** 1 Agroecosystem Sustainability Center, Institute for Sustainability, Energy, and Environment, University of Illinois Urbana-Champaign, Urbana, IL 61801 USA; 2 Department of Natural Resources and Environmental Sciences, College of Agricultural, Consumers, and Environmental Sciences, University of Illinois Urbana-Champaign, Urbana, IL 61801 USA; 3 DOE Center for Advanced Bioenergy and Bioproducts Innovation, Urbana, IL 61801 USA; 4 National Center of Supercomputing Applications, University of Illinois Urbana-Champaign, Urbana, IL 61801 USA; 5 Research Institute of Agriculture and Life Sciences, Seoul National University, Seoul, 08826 Republic of Korea; 6 Department of Environmental Sciences, University of Virginia, Charlottesville, VA 22903 USA

**Keywords:** Carbon cycle, Photosynthesis

## Abstract

Sun-induced chlorophyll fluorescence (SIF) provides an opportunity to study terrestrial ecosystem photosynthesis dynamics. However, the current coarse spatiotemporal satellite SIF products are challenging for mechanistic interpretations of SIF signals. Long-term ground SIF and vegetation indices (VIs) are important for satellite SIF validation and mechanistic understanding of the relationship between SIF and photosynthesis when combined with leaf- and canopy-level auxiliary measurements. In this study, we present and analyze a total of 15 site-years of ground far-red SIF (SIF at 760 nm, SIF_760_) and VIs datasets from soybean, corn, and miscanthus grown in the U.S. Corn Belt from 2016 to 2021. We introduce a comprehensive data processing protocol, including different retrieval methods, calibration coefficient adjustment, and nadir SIF footprint upscaling to match the eddy covariance footprint. This long-term ground far-red SIF and VIs dataset provides important and first-hand data for far-red SIF interpretation and understanding the mechanistic relationship between far-red SIF and canopy photosynthesis across various crop species and environmental conditions.

## Background & Summary

Chlorophyll fluorescence is the emission of light in the spectral range of 650–850 nm from the excited states of chlorophyll-a molecules in competition with photochemistry and heat dissipation^1^. It is tightly linked to photosynthesis from the molecular to canopy levels^2,3^. Detecting fluorescence is challenging due to its small percentage in the reflected radiance signal under natural sunlight (~1-2%)^4^. Pulse-amplitude modulated (PAM) fluorescence techniques with active light sources have long been used to induce fluorescence and are further used as a probe to study photosynthesis in the laboratory and natural fields^1,5^. However, active PAM measurements require close contact with leaves, which has limited its applications to the subcellular and leaf levels^6^. The feasibility of remotely detecting passive fluorescence, that is, sun-induced chlorophyll fluorescence (SIF), has extended the possibilities to monitor vegetation dynamics at the ecosystem, regional and global scales^7,8^. The first global terrestrial satellite SIF product was retrieved from meteorological satellites in 2011^9^. Afterward, a growing number of spaceborne SIF retrievals have been developed^10–13^, which have stimulated a wide range of SIF applications such as gross primary production (GPP) estimation^14–16^, crop productivity estimation^17–19^, and detection of various stress effects^20–22^.

These satellite technology developments have also spurred interest in ground remote sensing of SIF^23,24^. Ground SIF can facilitate the interpretation of SIF and its relationship with photosynthesis at leaf and canopy levels since satellite SIF usually have coarse spatial and limited temporal resolutions. Benchmark data of GPP is usually estimated from eddy covariance (EC) towers which measure the carbon, water vapor, and energy flux exchanges between terrestrial ecosystems and the atmosphere^25^. EC techniques capture ecosystem CO_2_ fluxes across a range of temporal scales from half hours to years, and samples footprints along the longitudinal dimensions ranging between a hundred meters and several kilometers depending on the tower setup, turbulent conditions, and underlying surface conditions^26^. Currently available satellite SIF products with coarse spatiotemporal resolution (e.g., 7 km × 3.5 km at nadir and nearly daily for TROPOspheric Monitoring Instrument (TROPOMI)) hinders the direct comparison between satellite SIF and ground GPP due to their sampling footprint mismatch. Ground remote sensing is capable of collecting SIF from minutes to days and sampling areas from several meters to hundreds of meters^27^, which is more comparable to ground GPP than satellite products. Therefore, ground SIF and vegetation indices (VIs) are crucial for bridging the measurement gap between flux measurements and satellite data. First, the high temporal resolution of ground SIF and VIs allows the investigation of diurnal relationships between SIF and GPP as well as their relationship under different environmental conditions^28–30^. Second, the spatial comparability between ground sensing and GPP is beneficial for exploring species-specific SIF-GPP relationships and mechanistic SIF-GPP relationships when combined with leaf-level measurements^31–34^. Additionally, ground sensing of SIF and VIs can be used as validation of satellite remote sensing products^35^. Various studies have shown the advantages of ground spectral measurements in connecting vegetation optical properties to EC flux measurements^36,37^.

Over the last several years, a number of spectral systems have been developed and deployed in the field for collecting automatic and continuous observations of canopy SIF and VIs, e.g., FluoSpec2^38^, FLOX (JB Hyperspectral Devices), Photospec^39^, FAME^40^, SIFSpec^41^ and SIFprism^42^. These systems are either bi-hemispherical systems that samples canopy radiance from 180° field of view (FOV) with the use of a cosine corrector (e.g., FAME and SIFprism), or hemispherical-directional systems which sample canopy radiance using a bare fiber with FOV ~25° (e.g., FluoSpec2 and Photospec). Each system is usually equipped with two spectrometers. One spectrometer with high-spectral resolution and signal-to-noise (SNR) ratio (e.g., QEPRO from Ocean Optics, Inc., Dunedin, FL, USA) is for SIF retrieval, and the other one covers visible to the near-infrared band for VIs estimation (e.g., HR2000 + from Ocean Optics). Ground spectral observations can be collected near EC towers to facilitate the direct investigation of SIF-VIs-GPP relationships and validation of satellite products. Numerous studies have investigated ground SIF and its relationship with stress and canopy photosynthesis, but most of them only focus on one single site, single growing season, and/or single species/ecosystem^31,32,43^. Additionally, although a few communities have integrated optical sampling with EC flux measurements, such as SpecNet (http://specnet.info)^44^, EUROSPEC (https://eurospec.eu)^37^ and ChinaSpec (http://chinaspec.nju.edu.cn)^45^, the SIF and concurrent VIs data availability is still limited across multiple years and sites.

In this paper, we present a dataset with 15 site-years of ground far-red SIF and VIs (including normalized difference vegetation index (NDVI), enhanced vegetation index (EVI), near-infrared of vegetation (NIRv), red edge chlorophyll index (CI_rededge_), green chlorophyll index (CI_green_) and photochemical reflectance index (PRI)) across multiple crop sites in the U.S. Corn Belt collected from 2016 to 2021. A FluoSpec2 system was used to collect the spectral data automatically and continuously in each growing season. The six VIs were chosen to reflect different aspects of terrestrial vegetation. Specifically, NDVI, EVI, and NIRv are mainly related to canopy structure, such as fraction of vegetation cover, leaf area index, and canopy architecture^46–48^. CI_rededge_ and CI_green_ are two widely used chlorophyll indices^49^, and PRI is related to the photosynthetic radiation use efficiency and non-photochemical quenching (NPQ)^50^. Sampled crop species include corn (C4 plant), soybean (C3 plant), and miscanthus (C4 plant). Corn and soybean are essential annual row crops which are widely used as human food, livestock feed, and raw materials in industry. Miscanthus is a promising perennial crop for bioenergy production attributed to its significant carbon sequestration, large biomass, and high nutrient use efficiency^51^. All the sites contain corresponding EC flux and meteorological data, which is beneficial for the direct exploration of SIF-VIs-GPP relationships across different crop species under different environmental conditions. The aim of this paper is to: (i) describe the instrumentation, data collection, and data processes for far-red SIF and VIs; and (ii) perform analyses of crop far-red SIF, VIs, as well as SIF-VIs relationships as an indirect validation of the dataset. The paper also aims to invite any researcher interested to proceed with further analysis of the data, which are made available in a public repository. The spectral system, field deployment, data collection and data processes, including different far-red SIF retrievals, radiometric calibration coefficient adjustments, upscaling nadir SIF to EC footprint, and VIs estimation, are described in Methods section. In Data Records and Technical Validation section, the retrieved far-red SIF, estimated VI, as well as the relationship between SIF and VIs across corn, soybean, and miscanthus, are presented.

## Methods

### Spectral system description

**Fig. 1:**
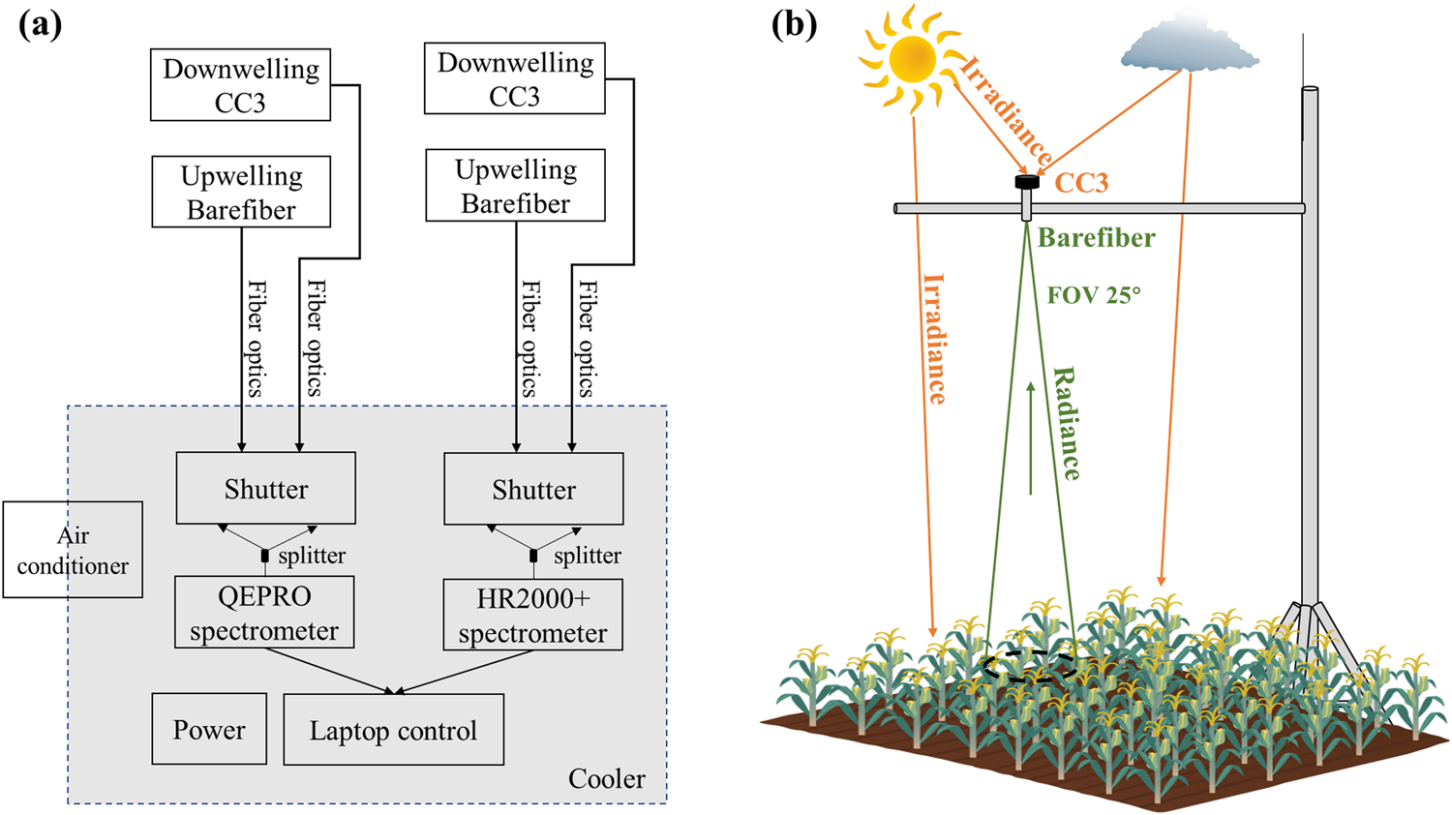
Schematic layout and deployment of FluoSpe2. (**a**) Schematic diagram of a FluoSpe2 system; (**b**) Conceptual field deployment of a FluoSpe2 system. FOV: field of view.

FluoSpec2, a hemispherical-directional system, was used for spectral data collection^38,52^. It consists of two paths, with each path equipped with one spectrometer, one splitting fiber, one inline shutter, and two fibers for downwelling irradiance and upwelling radiance collection, respectively (Fig. 1). The data collected by the two paths were used for far-red SIF retrieval and VIs estimation, respectively. For far-red SIF data collection, the spectrometer, QEPRO, covered wavelengths from 730–780 nm with a Full Width Half Maximum (FWHM) of 0.15 nm. For VIs estimation, the HR2000 + spectrometer with a wavelength coverage of 350–1100 nm and FWHM 1.1 nm was used (Ocean Optics). One cosine corrector (CC3, Ocean Optics) was attached to the irradiance fiber to achieve a FOV of 180° while a bare fiber with a FOV of 25° was installed at the nadir for canopy radiance collection. Two spectrometers were connected to a laptop to conduct automatic data collection. The spectral system, except the fibers, was placed in an enclosure with temperature controlled by an air conditioner. The target temperature was set to 25 °C. A temperature and humidity sensor (THC-4) was used to monitor the change in temperature and humidity continuously. Desiccant bags were added into the enclosure periodically to ensure the relative humidity (RH) was below 70%.

### Field system setups

**Fig. 2:**
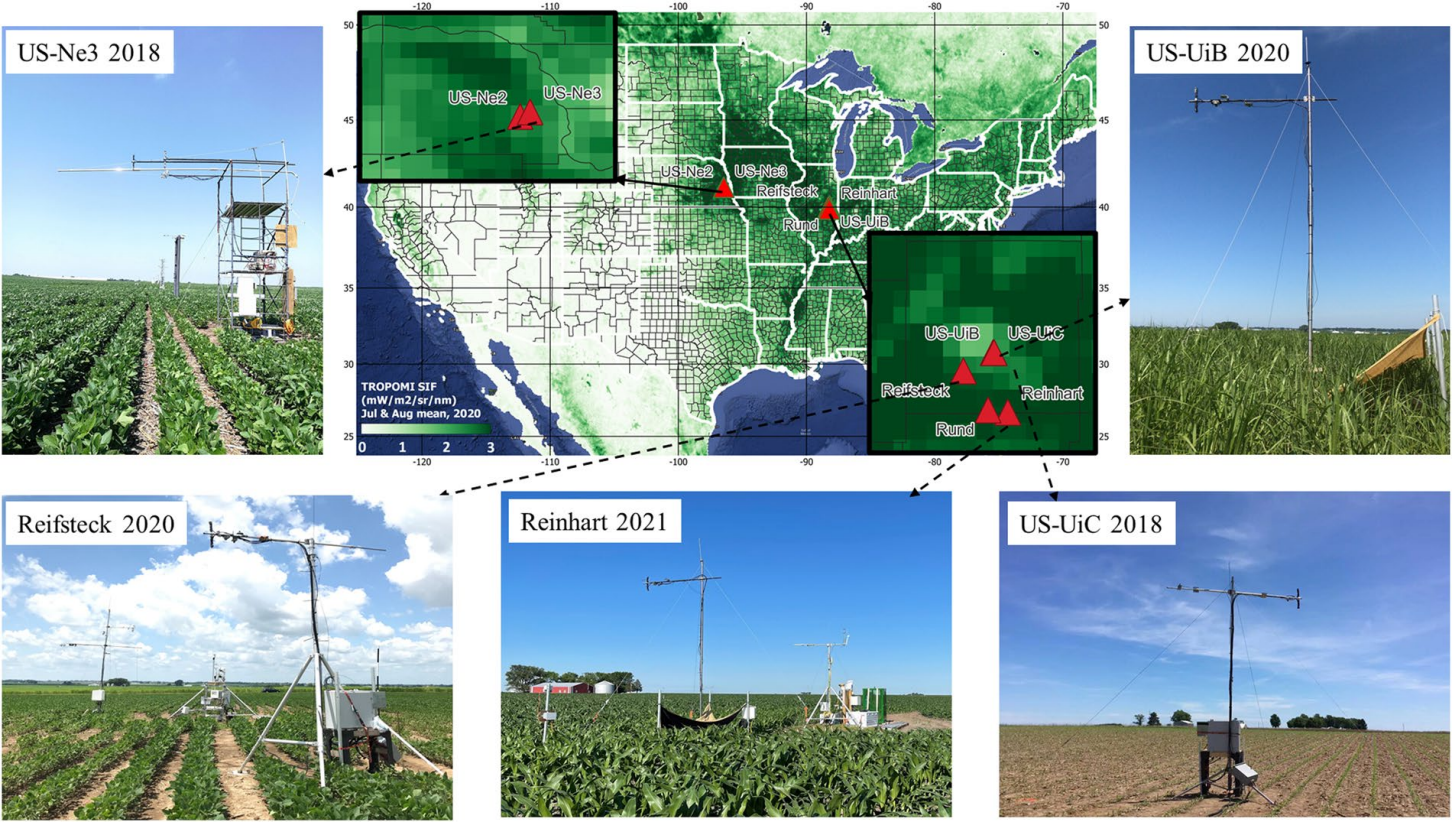
Field sites of our long-term ground measurements and some examples of field setups of FluoSpec2 systems.

At each site-year, the FluoSpec2 system was installed close to the EC tower to integrate with EC flux measurements. All of our sites were planted with one of the following crops: corn, soybean (Soy), or miscanthus (Mis). Considering that the maximum canopy height for those crops is below 3 m, a simple tripod with a bracket or a scaffold was used to hold the fibers at the height of 5 m, at which the spectral target area is ~2.2 meters in diameter on the ground (Fig. 2). When crops are fully grown, the spectral target area is around ~1.8 meters in diameter for soybean (maximum height ~ 1 m) and ~1.1 meters in diameter for corn and miscanthus (maximum height ~2.5 m). FluoSpec2 system was installed at seven sites in the U.S. Corn Belt near planting and uninstalled after harvest to collect whole growing-season data (Table 1). Two of the sites were in Lincoln, Nebraska (US-Ne2 and US-Ne3), and the other five sites were in Champaign, Illinois (US-UiB, US-UiC, Reifsteck, Rund, and Reinhart). Except for US-UiB where miscanthus emerged each year after the establishment in 2010, other sites were either corn-soybean rotation or corn-corn-soybean rotation. US-Ne2 was an irrigated site while other sites were rainfed. Fertilizers were applied for corn and miscanthus at all the sites. Detailed site information is summarized in Table 1. US-Ne2, US-Ne3, US-UiB, and US-UiC are registered on the AmeriFlux site (https://ameriflux.lbl.gov/), where EC and meteorological data can be freely downloaded. Reifsteck, Rund, and Reinhart sites are private farms, and EC and meteorological data can be obtained upon the request of PIs.

**Table 1:** Information of the field sites deployed with Fluospe2 systems. ‘‘Soy’’ refers to soybean and ‘‘Mis’’ refers to miscanthus.

Site	Latitude	Longitude	Crop type	Management
US-Ne2	41.1649°N	−96.4701°W	Corn-soy rotation	Irrigated, no-till, fertilizer applied for corn
US-Ne3	41.1797°N	−96.4397°W	Corn-soy rotation	Rainfed, no-till, fertilizer applied for corn
US-UiB	40.0628°N	−88.1984°W	Mis	Rainfed, N/A, fertilizer applied
US-UiC	40.0647°N	−88.1983°W	Corn-corn-soy rotation	Rainfed, conventional-till, fertilizer applied for corn
Reifsteck	39.8824°N	−88.1546°W	Corn-soy rotation	Rainfed, no-till, fertilizer applied for corn
Rund	40.0070°N	−88.2897°W	Corn-soy rotation	Rainfed, minimum-till, fertilizer applied for corn
Reinhart	39.8887°N	−88.2140°W	Corn-corn-soy rotation	Rainfed, conventional-till, fertilizer applied for corn

## Data collection

FluoSpec Manager, a software written in Visual Basic with libraries provided by Ocean Optics was installed on the laptop to control the automatic irradiance and radiance data collection at 5-minute intervals^38^. The integrating time for each spectrum was optimized by the algorithm in FluoSpec Manager with the target maximum digital number (DN) of 120000 for QEPRO and 12000 for HR2000+, respectively. For each 5-min interval, data was collected in the following sequence: HR2000+ irradiance – HR2000+ radiance – HR2000+ irradiance – QEPRO irradiance – QEPRO radiance – QEPRO irradiance. The dark current for QEPRO was collected after each observation with the same integrating time as the observation through controlling the internal shutter of QEPRO. For HR2000+, the dark signal was collected using OceanView (Ocean Optics) under various integrating times during the nighttime period, and the dark signal with a similar integrating time as the observation was used to match with each observation. From 2016 to 2021, a total of 15 site-years data were collected with eight site-years corn, five site-years soybeans, and two site-years miscanthus. For each site-year, corn and soybean were planted during April or May and harvested in September or October. Miscanthus emerged in March and was harvested in the following year in February or March. Detailed information about the data availability at each site-year is summarized in Table 2.

**Table 2:** Specific site information and spectral data availability of each year. ‘‘Soy’’ refers to soybean and ‘‘Mis’’ refers to miscanthus.

Site	Year	Crop	Growing season	SIF & Hyperspectral
US-Ne2	2017	Corn	May 8–Oct 30	Jul 15–Oct 15
	2018	Soy	May 14–Oct 19	Jun 19–Oct 14
US-Ne3	2017	Corn	May 8–Oct 30	Jul 15–Sep 17
	2018	Soy	May 14–Oct 19	Jul 8–Oct 14
	2019	Corn	Apr 27–Nov 6	May 3–Oct 15
US-UiB	2019	Mis	Apr 2019–Mar 2020	May 9–Nov 19
	2020	Mis	Apr 2020–Mar 2021	May 11–Nov 1
US-UiC	2016	Soy	May 27–Oct 17	Aug 7–Sep 24
	2017	Corn	May 16–Nov 2	Jun 7–Oct 29
	2018	Corn	May 8–Oct 9	Jun 28–Oct 10
	2019	Soy	May 17–Oct 9	Jun 5–Oct 6
Reifsteck	2020	Soy	Apr 21–Oct 3	May 2–Oct 2
	2021	Corn	May 1–Sep 26	May 16–Sep 11
Rund	2021	Corn	Apr 26–Dec 2	May 30–Sep 18
Reinhart	2021	Corn	Apr 23–Sep 25	May 15–Sep 21

In-field radiometric calibration was conducted on all the fibers connected to the upward cosine corrector and downward bare fiber when FluoSpec2 was assembled. First, A homogenous light source with known intensity (a tungsten–halogen light source, HL-3P-CAL, Ocean Optics) was used to calibrate the upward cosine corrector through the OceanView “absolute irradiance” module. Second, for the downward bare fiber pointing to the canopy, a cross-validation method was used. Specifically, when the skies were sunny and the solar zenith angle was not high (local time between 10 am to 3 pm), the four fibers were installed in a way that the calibrated cosine correctors pointed to the sky, and the downward bare fibers pointed to a spectralon panel with known reflectance (Labsphere, Inc., NH, USA) at the same time; then the calibrated irradiance path was used to cross-calibrate the radiance path. No shadow on the spectralon panel was allowed in the footprint of the downward bare fibers when conducting the calibration. At least three times in-field calibrations were conducted during the growing season. The calibration coefficients for HR2000+ were stable across the whole growing season while for QEPRO they showed variations. The calibration coefficient for which the retrieved far-red SIF value was closest to zero for all the collected spectralon panels’ data was used to obtain absolute irradiance and radiance for QEPRO and HR2000+ based on the assumption that spectralon panels did not emit fluorescence.

**Fig. 3:**
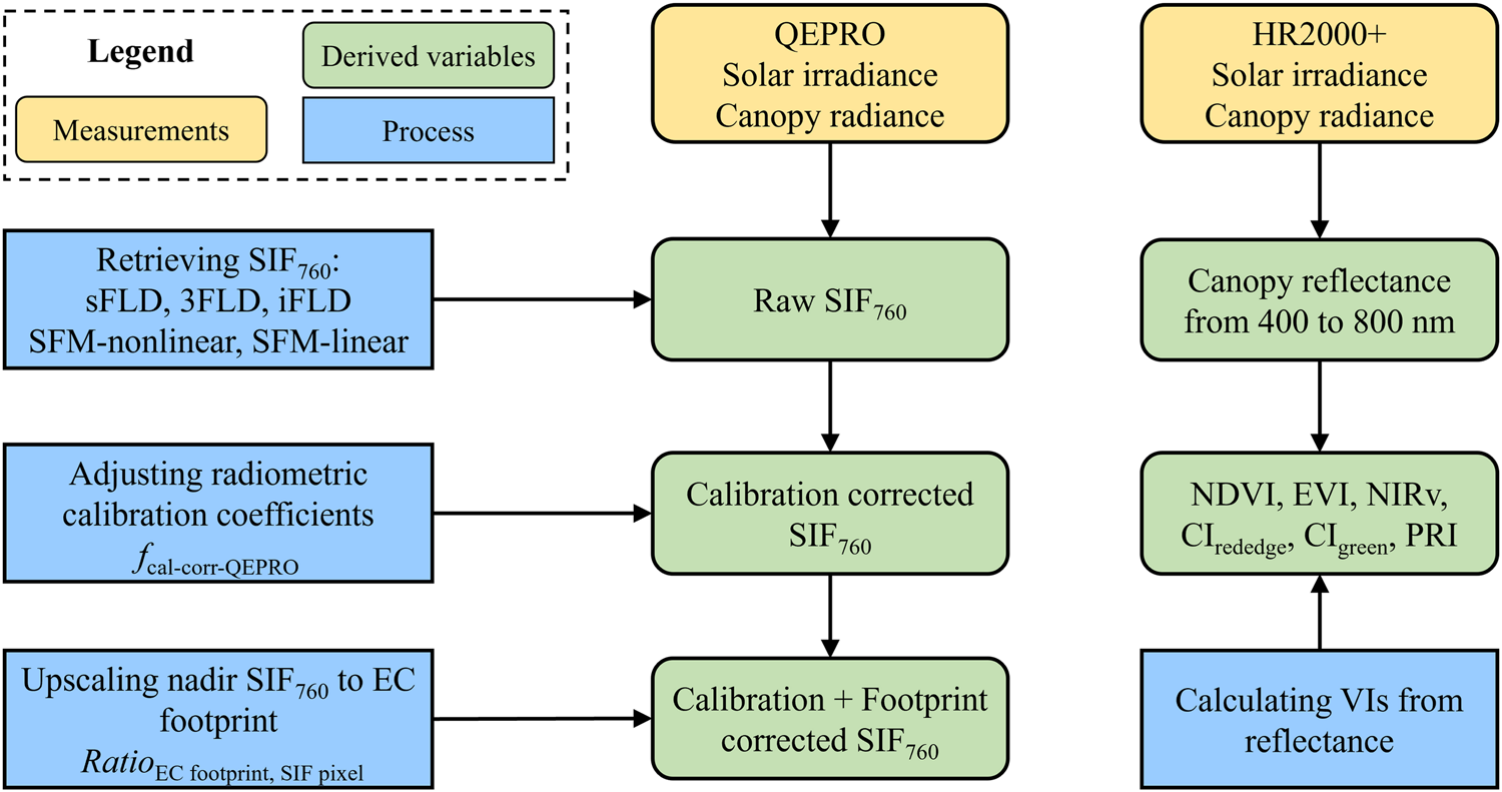
Flowchart of data processing at each site-year. sFLD: standard Fraunhofer line depth; 3FLD: three-band Fraunhofer line depth; iFLD: improved Fraunhofer line depth; SFM-nonlinear: spectral fitting method with the assumption of non-linear variation of fluorescence and reflectance over the absorption band; SFM-linear: spectral fitting method with the assumption of linear variation of fluorescence and reflectance over the absorption band; *f*_cal-corr-QEPRO_: the calibration adjustment factor for SIF; EC: eddy covariance; $Rati{o}_{ECfootprint,SIFpixel}$: the ratio between EC footprint weighted VI and SIF tower located pixel VI.

Collected solar irradiance and canopy radiance data from QEPRO and HR2000+ were used for far-red SIF retrieval and VIs estimation, respectively. For each site year, different SIF retrieval algorithms were first used to derive raw SIF at 760 nm (SIF_760_). Radiometric calibration coefficients were then adjusted to account for the calibrating light source degradation across years. Calibration-corrected SIF_760_ was finally upscaled to match the EC footprint. Different VIs were estimated from the visible to near-infrared band reflectance calculated from HR2000+ irradiance and radiance. Considering the large uncertainty of SIF_760_ data under low light conditions, only SIF_760_ and VIs data collected from local time 8 am to 6 pm when the solar zenith angle was smaller than 90° were used. A flowchart of data processing at each site-year is summarized in Fig. 3. The processed half-hourly SIF_760_ and VIs are available at the on Oak Ridge National Laboratory Distributed Active Archive Center (ORNL DAAC) data repository 10.3334/ORNLDAAC/2136^53^.

### SIF retrieval methods

SIF adds a weak signal to the reflected solar radiation, which results in two contributions to the upwelling radiance (L). Most retrieval algorithms for ground SIF are based on the Fraunhofer line depth (FLD) principle and the spectral fitting method (SFM). FLD approaches exploit the reduced downwelling irradiance (E) inside the oxygen absorption bands (O_2_A and O_2_B) reaching the surface, which results in an increase in the relative contribution of SIF to L. Several FLD methods are commonly used in ground SIF retrieval, including standard FLD (sFLD), three bands FLD (3FLD), and improved FLD (iFLD). All of them rely on the measurements of E and L inside and outside the absorption window (λ_in_ and λ_out_). Here, we specifically focus on the O_2_A absorption window (760 nm) considering the wavelength coverage of QEPRO. The upwelling radiance inside and outside the O_2_A band (L(λ_in_), L(λ_out_)) is a function of reflectance (R(λ_in_), R(λ_out_)), irradiance (E(λ_in_), E(λ_out_)) and SIF (SIF(λ_in_), SIF(λ_out_)) inside and outside the absorption band, respectively:

1 $$L\left({\lambda }_{in}\right)=\frac{R\left({\lambda }_{in}\right)\times E\left({\lambda }_{in}\right)}{\pi }+SIF\left({\lambda }_{in}\right),$$

2 $$L\left({\lambda }_{out}\right)=\frac{R\left({\lambda }_{out}\right)\times E\left({\lambda }_{out}\right)}{\pi }+SIF\left({\lambda }_{out}\right),$$

For sFLD which assumes that the R and SIF are the same inside and outside the absorption band ($R\left({\lambda }_{in}\right)=R\left({\lambda }_{out}\right)$), $SIF\left({\lambda }_{in}\right)=SIF\left({\lambda }_{out}\right)$), SIF can be derived as follows:

3 $$SI{F}_{SFLD}=\frac{E\left({\lambda }_{out}\right)\times L\left({\lambda }_{in}\right)-E\left({\lambda }_{in}\right)\times L\left({\lambda }_{out}\right)}{E\left({\lambda }_{out}\right)-E\left({\lambda }_{in}\right)},$$

As a more advanced method than sFLD, 3FLD assumes that R and F change linearly over the absorption window. Therefore, the single reference outside band used in sFLD (λ_out_) is replaced by the average of two bands of the left and right shoulders of the absorption line. However, non-linear variation of R and SIF could result in inaccurate SIF estimates. Therefore, iFLD uses two correction factors (*α*_*R*_ and *α*_*F*_) to account for the non-linear change of R and F over the absorption window^54^. Instead of two or three bands, iFLD utilizes the whole R and E spectral information to estimate *α*_*R*_ and *α*_*F*_. Specifically, they are estimated by the apparent reflectance (*R*_*app*_) which is contaminated with fluorescence signal as follows:

4 $${\alpha }_{R}\approx \frac{{R}_{app}\left({\lambda }_{out}\right)}{{R}_{app}\widetilde{\left({\lambda }_{in}\right)}},$$

5 $${\alpha }_{F}\approx \frac{E\left({\lambda }_{out}\right)}{E\widetilde{\left({\lambda }_{in}\right)}},$$

where *R*_*app*_(*λ*_*out*_) is the apparent reflectance outside the absorption band, and ${R}_{app}\widetilde{\left({\lambda }_{in}\right)}$ is the apparent reflectance inside the absorption band which is obtained from the non-linear interpolation of the apparent reflectance using the continuouss reflectance spectrum at the left and right shoulders. Analogously, $E\widetilde{\left({\lambda }_{in}\right)}$ is obtained by the interpolation of the irradiance. SIF is calculated as follows:

6 $$SI{F}_{iFLD}=\frac{{\alpha }_{R}\times E\left({\lambda }_{out}\right)\times L\left({\lambda }_{in}\right)-E\left({\lambda }_{in}\right)\times L\left({\lambda }_{out}\right)}{{\alpha }_{R}\times E\left({\lambda }_{out}\right)-{\alpha }_{F}\times E\left({\lambda }_{in}\right)},$$

Different from FLD-based approaches, SFM method aims to decouple SIF and reflectance from radiance observations through general mathematical representations of canopy SIF and R within the narrow absorption windows centered at 760 nm. The parameterization of functions for SIF and R is optimized by the least-square optimization process with observed radiance as a reference. Both linear and non-linear functions can be used to represent SIF and R. Here, we tried both the linear method, which assumed that SIF and R both linearly changed with wavelength^52^, and the non-linear method, for which a Gaussian function was used to model SIF and a cubic spline function was used to model R^55^. For the linear model:

7 $$L=\frac{R\times E}{\pi }+SIF,$$

8 $$R=a\times \lambda +b,$$

9 $$SIF=c\times \lambda +d,$$

For the non-linear model, R was approximated by a cubic spline function and SIF was modelled as follows:

10 $$SIF=a{\prime} \times {e}^{-\frac{{\left(\left(\lambda -{\lambda }_{0}\right)-\left(c{\prime} -{\lambda }_{0}\right)\right)}^{2}}{2\times {b{\prime} }^{2}}}$$

Parameters a, b, c, d of the linear method and a′, b′ and c′ of the non-linear method were optimized to match the observed L. When *λ* is set as 760 nm, SIF_760_ is estimated. The values of these five-method estimated SIF_760_ were all presented as well as their comparison. SIF_760_ was retrieved at the raw 5-minute interval. SIF_760_ values below 0 or above 5 were discarded as outliers. Raw 5-minute SIF_760_ was averaged to half-hourly timestamp to match the EC data when more than four data points were available during the half-hourly period. The standard error of 5-minute SIF_760_ within the half hour was regarded as the uncertainty of each method retrieved SIF_760_. Detailed information about the selection of wavelength outside the absorption feature, as well as the absorption windows, can be found in^55^.

### Radiometric calibration coefficient adjustment for SIF

**Fig. 4:**
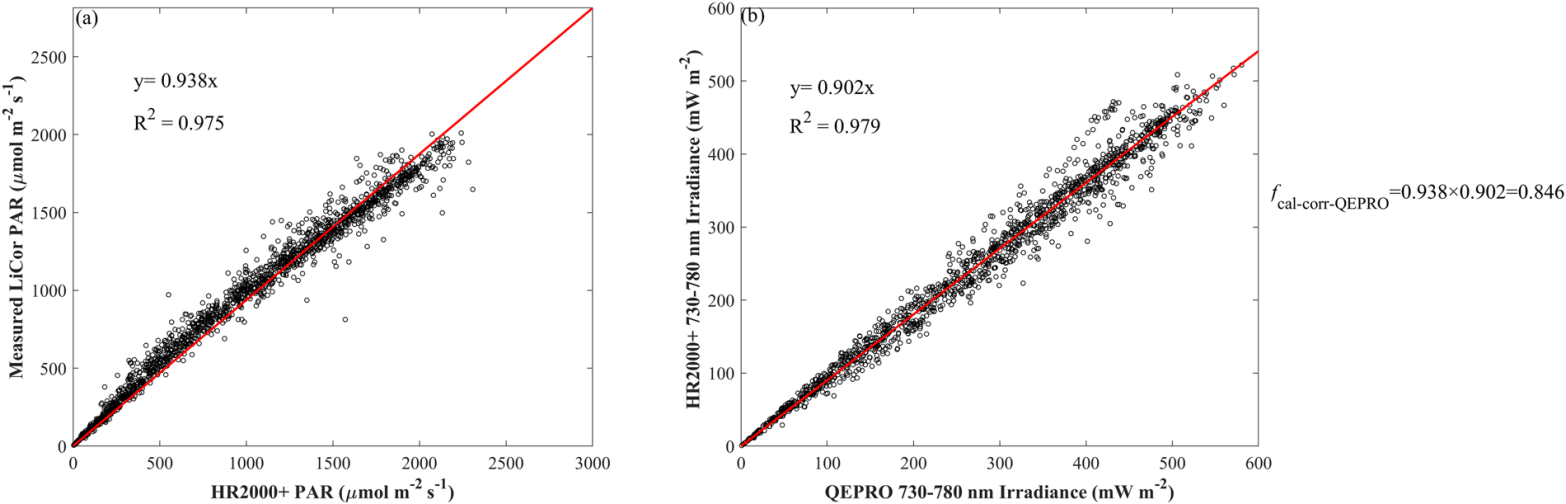
An example showing the calculation of the calibration adjustment factor for SIF_760_ (*f*_cal-corr-QEPRO_) at US-Ne2 2017 corn. (**a**) the relationship between PAR calculated from HR2000 + spectrometer and measured PAR from LiCor quantum sensor; (**b**) the relationship between near-infrared irradiance integrated from 730 nm calculated from QEPRO spectrometer and that from HR2000 + . Red lines are fitted linear regression lines without intercept.

**Fig. 5:**
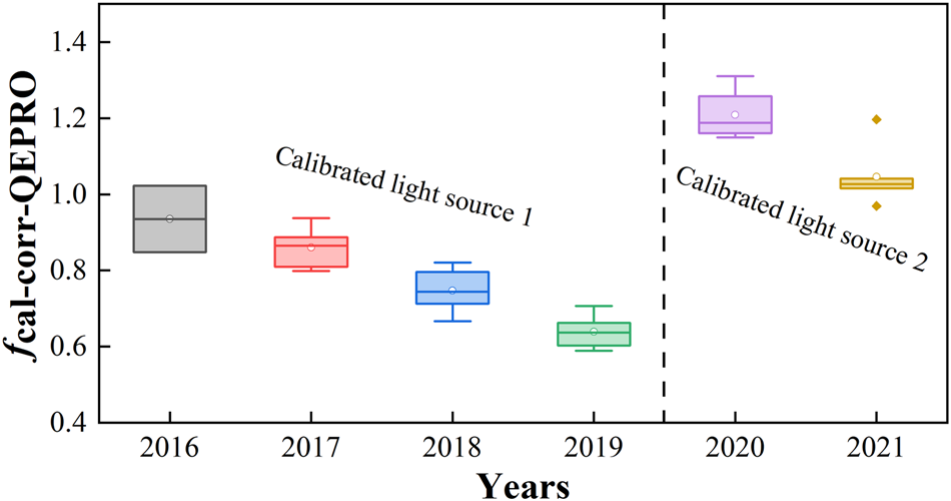
The variation of the calibration adjustment factor for SIF_760_ (*f*_cal-corr-QEPRO_) from 2016 to 2021. The first calibrated light source is used for irradiance calibration from 2016 to 2019, and the second one is used from 2020 to 2021.

To account for the degradation of the light source used for irradiance calibration, a cross-calibration method was used to adjust the change of radiometric calibration coefficients across years. Although the light source could be used for 50 hours based on the manufacturer, we still noticed a pattern of degradation across the years within 50 hours. This light source signal degradation would affect the estimation of SIF_760_ since SIF_760_ is an absolute light signal, while it does not affect the calculation of VIs since VIs are derived from reflectance, which is a ratio. To adjust for the degradation effect, for each site-year, we first calculated the photosynthetic active radiation (PAR) from HR2000+ by integrating the irradiance from 400 to 700 nm, then we compared HR2000+ -based PAR with a LiCor quantum sensor that was well calibrated, from which a first correction factor was obtained. Second, we compared the near-infrared irradiance from 730 to 780 nm between QEPRO and HR2000+, from which a second corrector factor was obtained. Last, the product of the first correction factor and the second correction factor was used as the final radiometric calibration coefficient adjustment factor for QEPRO (*f*_cal-corr-QEPRO_). Figure 4 shows an example of how to obtain *f*_cal-corr-QEPRO_ at US-Ne2 2017 corn. Figure 5 shows the interannual variation of *f*_cal-corr-QEPRO_ from 2016 to 2019 with the first light source and from 2020 to 2021 with the second one, from which an obvious degradation pattern is observed, indicated by the further increase in *f*_cal-corr-QEPRO_ deviating from 1 with the used year increase. Calibration-corrected SIF_760_ was obtained by multiplying this *f*_cal-corr-QEPRO_ to the retrieved raw SIF_760_.

### Footprint upscaling for *in-situ* nadir SIF to match GPP footprint

Considering that EC footprint covers a larger area compared to nadir SIF_760_ and that EC footprint changes with wind direction while SIF_760_ target area is fixed during the data collection, we propose a method to upscale nadir SIF_760_ to EC footprint through high spatiotemporal satellite VIs. The rational base for this upscaling is that the product of NIRv and PAR could explain the majority of the spatiotemporal variations in SIF_760_^56,57^. PlanetScope satellite provides the surface reflectance at daily timestamp with a 3 m spatial resolution^58^, from which daily NIRv was calculated and used for upscaling SIF_760_ to EC footprint. To further reduce the soil background impact on NIRv, soil adjusted NIRv (SANIRv) was further calculated following the method in^59^ and used for SIF_760_ footprint upscaling. EC footprint models were run at half-hourly timestamps to obtain the footprint weights (w_i_) of each 3 m × 3 m pixel within 2 km × 2 km centered at the EC tower. SIF_760_ tower location was represented by one 3 m × 3 m pixel. The upscaled SIF_760_ was calculated based on the following equations:

11 $$SANIR{v}_{ECfootprint}=\mathop{\sum }\limits_{i=1}^{N}{w}_{i}\times SANIR{v}_{i},$$

12 $$Rati{o}_{ECfootprint,SIFpixel}=\frac{SANIR{v}_{ECfootprint}\times PA{R}_{ECfootprint}}{SANIR{v}_{SIFpixel}\times PA{R}_{SIFpixel}}\approx \frac{SANIR{v}_{ECfootprint}}{SANIR{v}_{SIFpixel}},$$

13 $$SI{F}_{ECfootprint}=SI{F}_{nadir}\times Rati{o}_{ECfootprint,SIFpixel},$$

where *SANIRv*_*EC footprint*_ is calculated by the sum of the product of SANIRv ($SANIR{v}_{i}$) and footprint weight (w_i_) at each pixel i across all the pixels within the EC footprint N. *SANIRv*_*SIF pixel*_ is the SANIRv value at the SIF tower located pixel. With the assumption that PAR did not vary within the EC footprint, i.e., $PA{R}_{ECfootprint}=PA{R}_{ECfootprint}$, $Rati{o}_{ECfootprint,SIFpixel}$ was calculated as the ratio of $SANIR{v}_{ECfootprint}$ to $SANIR{v}_{SIFpixel}$. This method also assumed that far-red fluorescence yield did not change within the EC footprint. The Simple Analytical Footprint model on Eulerian coordinates (SAFE) developed by^60^ was used to calculate the EC footprint weights. This upscaling was not conducted at the US-UiC 2016 soybean and US-Ne3 2019 corn sites due to the unavailability of PlanetScope data in 2016 and the missing inputs for the EC footprint model at the US-Ne3 2019 corn site. More details about the footprint upscaling process and related uncertainties can be found in Wu *et al*.^61^.

### Vegetation indices estimation and SIF decomposition analysis

Several commonly used VIs including NDVI, EVI, NIRv, CI_rededge_, CI_green_ and PRI were estimated from the hyperspectral reflectance collected by HR2000+. The reflectance beyond 800 nm was noisy, therefore, reflectance from 770 to 780 nm was used as the near-infrared reflectance. The equations for VIs calculation were shown as follows:

14 $$NDVI=\frac{{R}_{770-780}-{R}_{650-660}}{{R}_{770-780}+{R}_{650-660}},$$

15 $$NIRv={R}_{770-780}\times NDVI,$$

16 $$EVI=2.5\times \frac{{R}_{770-780}-{R}_{650-660}}{{R}_{770-780}+6\times {R}_{650-660}-7.5\times {R}_{460-470}+1},$$

17 $$C{I}_{rededge}=\frac{{R}_{770-780}}{{R}_{720-730}}-1,$$

18 $$C{I}_{green}=\frac{{R}_{770-780}}{{R}_{545-565}}-1,$$

19 $$PRI=\frac{{R}_{531}-{R}_{570}}{{R}_{531}+{R}_{570}},$$

Raw 5-minute reflectance (R) was first averaged to half-hourly timestamp and then used for VIs calculation in order to obtain half-hourly VIs.

Based on the light use efficiency framework, SIF can be decomposed into fraction of absorbed photosynthetic active radiation (fPAR), PAR, fluorescence yield of the canopy (Φ_F, canopy_), escape probability from the canopy (f_esc_), as demonstrated in Eq. (20):

20 $$SIF=fPAR\times PAR\times {{\Phi }}_{F,canopy}\times {f}_{esc}$$

fPAR at most of the site-years were derived from *in situ* PAR measurements, except US-UiC 2016 soybean, US-UiC 2017 corn and US-UiC 2018 corn. Specifically, incoming PAR (PAR_in_) and surface reflected PAR (PAR_out_) were measured by point quantum sensors (LI-190; LICOR Bioscience, NE, USA). Transmitted PAR (PAR_trans_) was measured by line quantum sensors (LI-191; LICOR Bioscience) placed about 2 cm above the ground. fPAR_Meas_ and APAR_Meas_ were derived as follows:

21 $$fPA{R}_{Meas}=\frac{PA{R}_{in}-PA{R}_{out}-PA{R}_{trans}}{PA{R}_{in}},$$

22 $$APA{R}_{Meas}=fPA{R}_{Meas}\times PA{R}_{in},$$

For US-Ne2 and US-Ne3, PAR reflected by soil (PAR_soil_) was additionally measured by line quantum sensors facing downward. Therefore, for those two sites, fPAR_Meas_ was calculated as:

23 $$fPA{R}_{Meas}=\frac{PA{R}_{in}-PA{R}_{out}-PA{R}_{trans}+PA{R}_{soil}}{PA{R}_{in}},$$

For the three site-years without PAR_trans_ measurements, fPAR was estimated by the red edge normalized difference vegetation index (Rededge NDVI):^32,52,62^

24 $$fPA{R}_{VI}=1.37\times Rededge\,NDVI-0.17,$$

25 $$Rededge\,NDVI=\frac{{R}_{775}-{R}_{708}}{{R}_{775}+{R}_{708}},$$

26 $$APA{R}_{VI}=fPA{R}_{VI}\times PA{R}_{in},$$

This VI method for fPAR calculation was not applied at the miscanthus site since it was developed for corn and soybean.

f_esc_ was estimated by fPAR and NIRv proposed by Zeng *et al*.^63^:

27 $${f}_{esc}=\frac{NIRv}{fPAR},$$

Φ_F, canopy_ was derived from the following equation:

28 $${{\Phi }}_{F,canopy}=\frac{SIF}{fPAR\times PAR\times {f}_{esc}},$$

We quantified the contributions of fPAR, PAR, Φ_F, canopy,_ and f_esc_ to the variations of SIF using the relative importance method proposed by Lindeman, Merenda, and Gold (LMG)^64,65^ which decomposed the determination coefficient of linear regression (R^2^) to the contributions of each regressor. Considering the different fPAR estimation methods as well as different data availability of each site-year across the growing season (Table 2), we only focused on the peak growing season which was defined as the period when NDVI was larger than 85% of the maximum NDVI for each site-year across the growing season. The relationship between SIF_760_ and each VI as well as between SIF_760_ and the product of PAR and each VI were investigated for each species. The daytime average of SIF_760_ and VIs were calculated on days when more than 75% percent of the half-hourly data were available from 8 am to 6 pm local time.

## Data Records

The entire dataset is saved in one *csv* file with data gathered from 2016 to 2021 and is available at the on ORNL DAAC data repository 10.3334/ORNLDAAC/2136^53^. This dataset is openly shared, without restriction, in accordance with the Earth Observing System Data and Information System (EOSDIS) Data Use and Citation Policy (https://daac.ornl.gov/about/#citation_policy). Each row of the *csv* is an observation, and each column is a variable. The full dataset (*SIF_VegIndices_Illinois_Nebraska_Halfhour.csv*) has 37501 rows and 32 columns with the variable name shown at the first row.

- site: sites where the data was collected, as shown in Table 1.
- year: the year when the data was collected.
- species: the crop type of the site-year.
- latitude: the latitude of the site.
- longitude: the longitude of the site.
- timestamp_start: the start date and time of each data record shown as US Central Standard Time (CST).
- timestamp_end: the end date and time of each data record shown as US Central Standard Time (CST).
- doy: the day of year of each data record.
- SIF_sFLD_raw: the raw SIF_760_ retrieved from irradiance and radiance using the sFLD method with unit mw m^−2^ nm^−1^ sr^−1^.
- SIF_sFLD_raw_stderror: the standard error of sFLD-retrieved SIF_760_.
- SIF_3FLD_raw: the raw SIF_760_ retrieved from irradiance and radiance using the 3FLD method with unit mw m^−2^ nm^−1^ sr^−1^.
- SIF_3FLD_raw_stderror: the standard error of 3FLD-retrieved SIF_760_.
- SIF_iFLD_raw: the raw SIF_760_ retrieved from irradiance and radiance using the iFLD method with unit mw m^−2^ nm^−1^ sr^−1^.
- SIF_iFLD_raw_stderror: the standard error of iFLD-retrieved SIF_760_.
- SIF_SFM_nonlinear_raw: the raw SIF_760_ retrieved from irradiance and radiance using the SFM method and non-linear assumption with unit mw m^−2^ nm^−1^ sr^−1^.
- SIF_SFM_nonlinear_raw_stderror: the standard error of SFM-retrieved SIF_760_ with non-linear assumption.
- SIF_SFM_linear_raw: the raw SIF_760_ retrieved from irradiance and radiance using the SFM method and linear assumption with unit mw m^−2^ nm^−1^ sr^−1^.
- SIF_SFM_linear_raw_stderror: the standard error of SFM-retrieved SIF_760_ with linear assumption.
- f_cal_corr_QEPRO: the radiometric calibration adjustment factor for SIF_760_
- ratio_Ecfootprint_SIFpixel: the ratio EC footprint weighted SANIRv to SIF tower pixel SANIRv
- PAR: PAR measured by quantum sensor with unit umol m^−2^ s^−1^.
- FPAR_VI: FPAR calculated by Rededge NDVI.
- APAR_VI: the product of FPAR_VI and PAR with unit umol m^−2^ s^−1^.
- FPAR_measured: measured FPAR using quantum sensors.
- APAR_measured: the product of FPAR_measured and PAR with unit umol m^−2^ s^−1^.
- NDVI: normalized difference vegetation index
- EVI: enhanced vegetation index
- NIRv: near-infrared reflectance of vegetation
- CI_red_edge: red edge chlorophyll index
- CI_green: green chlorophyll index
- PRI: photochemical reflection index
- enclosure_temp: the temperature of the enclosure where the spectral system was located with unit °C.

Each data record is shown at half-hourly timestamp. −9999 is filled when no record is available.

## Technical Validation

The following subsections show quality validation of the dataset^53^. Since direct validation of ground SIF_760_ is not available, we characterized the quality of SIF_760_ by comparing SIF_760_ from different methods, comparing the seasonal variations of SIF_760_ and VIs at each site-year, comparing the peak-season SIF_760_ and VIs magnitude across different species, comparing the relationship between SIF_760_ and different VIs, and decomposing peak-season SIF_760_ into structural (fPAR), radiation (PAR) and physiological (${{\Phi }}_{F,canopy}$) components.

### Comparison of SIF_760_ retrievals from different methods

**Fig. 6:**
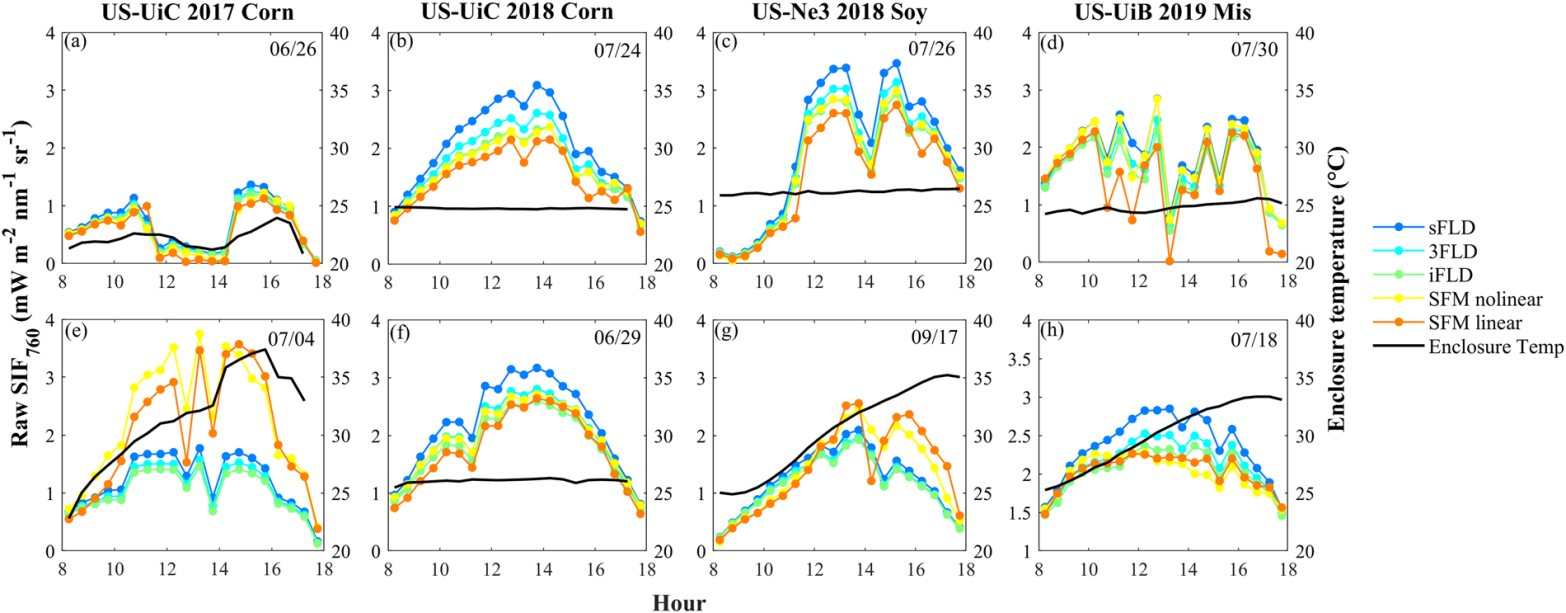
The diurnal variations of retrieved SIF_760_ from five methods (colored lines) and enclosure temperature (black lines) at eight representative days. The upper panel represents days when enclosure temperatures are well controlled, while the bottom panel represents days when enclosure temperatures fluctuate substantially except for US-UiC 2018 when enclosure temperature is well controlled across the whole data period.

The enclosure temperatures at some site-years were not well controlled at 25 °C due to the high summer temperatures at our sites (air temperature up to 35 °C); therefore, we specifically compared the SIF retrievals under different enclosure temperatures. Four representative site-years were selected to cover the three species as well as different enclosure temperature ranges: US-UiC 2017 corn, US-UiC 2018 Corn, US-Ne3 2018 soy, and US-UiB 2019 Mis. When the enclosure temperature was well controlled at around 25 °C, the five methods retrieved SIF_760_ showed similar diurnal patterns although sFLD and 3FLD retrieved SIF_760_ tended to show higher values, and SFM retrieved SIF_760_ showed the lowest values compared to other methods under sunny days (Fig. 6a-d,f). However, on days when the enclosure temperature was above 25 °C, for corn and soybean, the two SFM methods retrieved SIF_760_ increased with the increase of enclosure temperature in the afternoon (Fig. 6e,g), and this pattern was not obvious in miscanthus, possibly due to the lower enclosure temperature compared to the US-UiC 2017 corn and US-Ne3 2019 soybean.

**Fig. 7:**
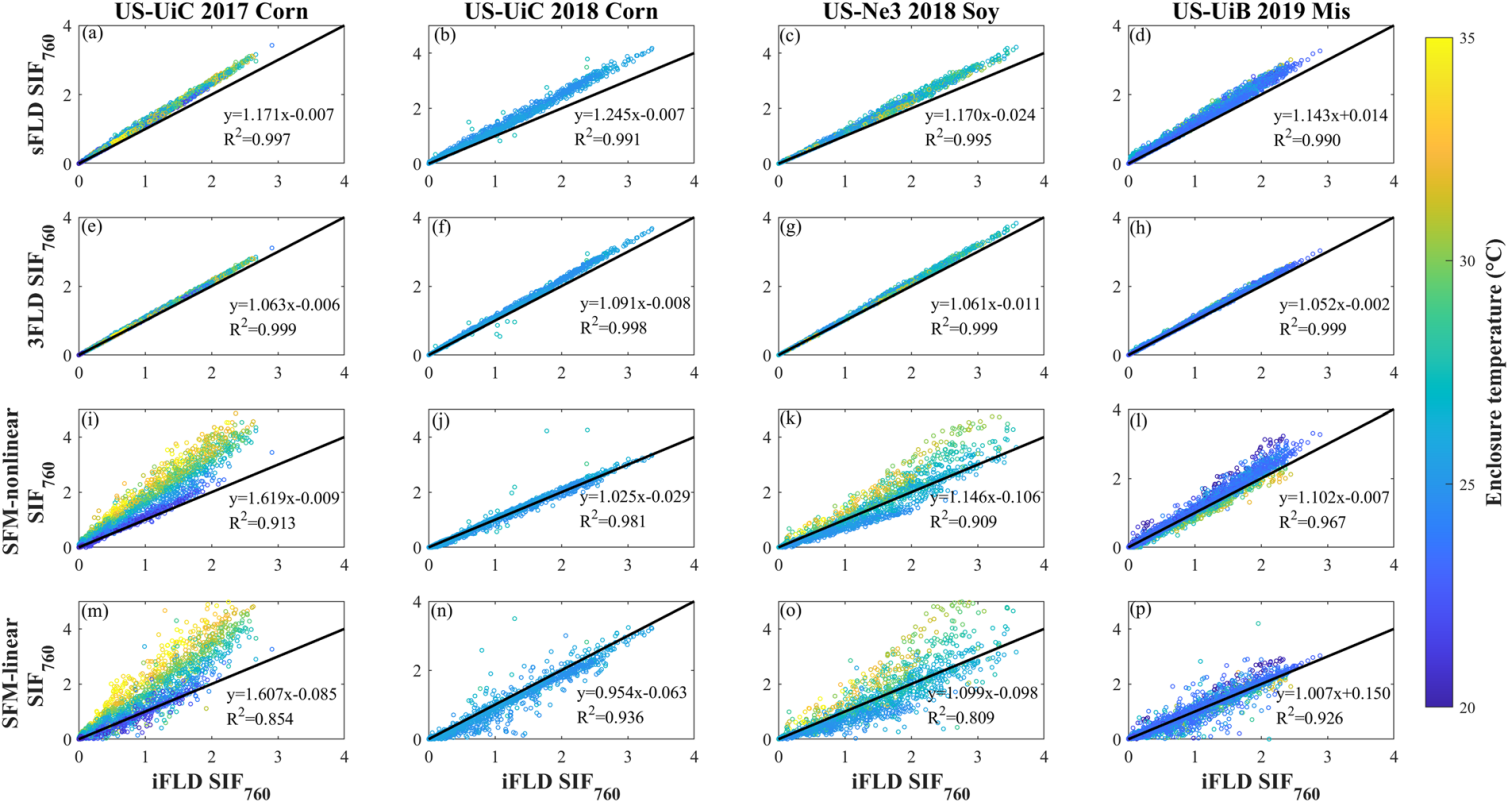
The relationship between different method retrieved SIF_760_ under different enclosure temperatures. The relationship between iFLD SIF_760_ and sFLD SIF_760_ (first row), between iFLD SIF_760_ and 3FLD SIF_760_ (second row), between iFLD SIF_760_ and SFM-nonlinear SIF_760_ (third row), and between iFLD SIF_760_ and SFM-linear SIF_760_ (fourth row) at US-UiC 2017 corn (first column), US-UiC 2018 corn (second column), US-Ne3 2018 soy (third column) and US-UiB 2019 Mis (fourth column). Colormap represents enclosure temperature. Black lines are 1:1 line.

At the seasonal scale, five methods retrieved SIF_760_ were strongly correlated with each other with R^2^ above 0.93 when the enclosure temperature was overall well controlled at around 25 °C, e.g., US-UiC 2018 corn and US-UiB 2019 miscanthus (Fig. 7), which indirectly demonstrated the reliability of our retrieved SIF_760_^53^. At the site-years with enclosure temperature reaching above 30 °C, e.g., US-UiC 2017 corn and US-Ne3 2018 soybean, FLD-based SIF_760_ were still strongly correlated with each other. However, SFM-based SIF_760_ increased with enclosure temperature which degraded the relationship between SFM-based SIF_760_ and iFLD-based SIF_760_ (Fig. 7i,m,k,o). SFM-based SIF_760_ with linear assumptions of SIF and R always showed the lowest correlation with other methods-based SIF_760_, indicating that linear assumptions of SIF and R might bring more uncertainties in SIF_760_ retrieval compared to other methods. Additionally, these results demonstrated that SFM-based methods were more sensitive to enclosure temperature compared to FLD-based methods, which can be explained by their algorithms. For FLD-based methods, both irradiance and radiance were used for SIF_760_ calculations as numerators and denominators. Enclosure temperature seemed to have similar effects on the spectrum shifts of irradiance and radiance, which cancelled each other when estimating SIF_760_ (Eqs. 3–6). However, for SFM-based methods, only the radiance and reflectance spectrums were used for fitting, therefore the spectral shift of radiance caused by enclosure temperature directly affected the SIF_760_ estimation. Based on these results, we recommend using FLD-based SIF_760_ when the enclosure temperature is not stable. Considering that iFLD method is more advanced compared to sFLD and 3FLD, iFLD-based SIF_760_ was used for further validation analysis.

### Variations of SIF_760_ in corn, soybean and miscanthus

**Fig. 8:**
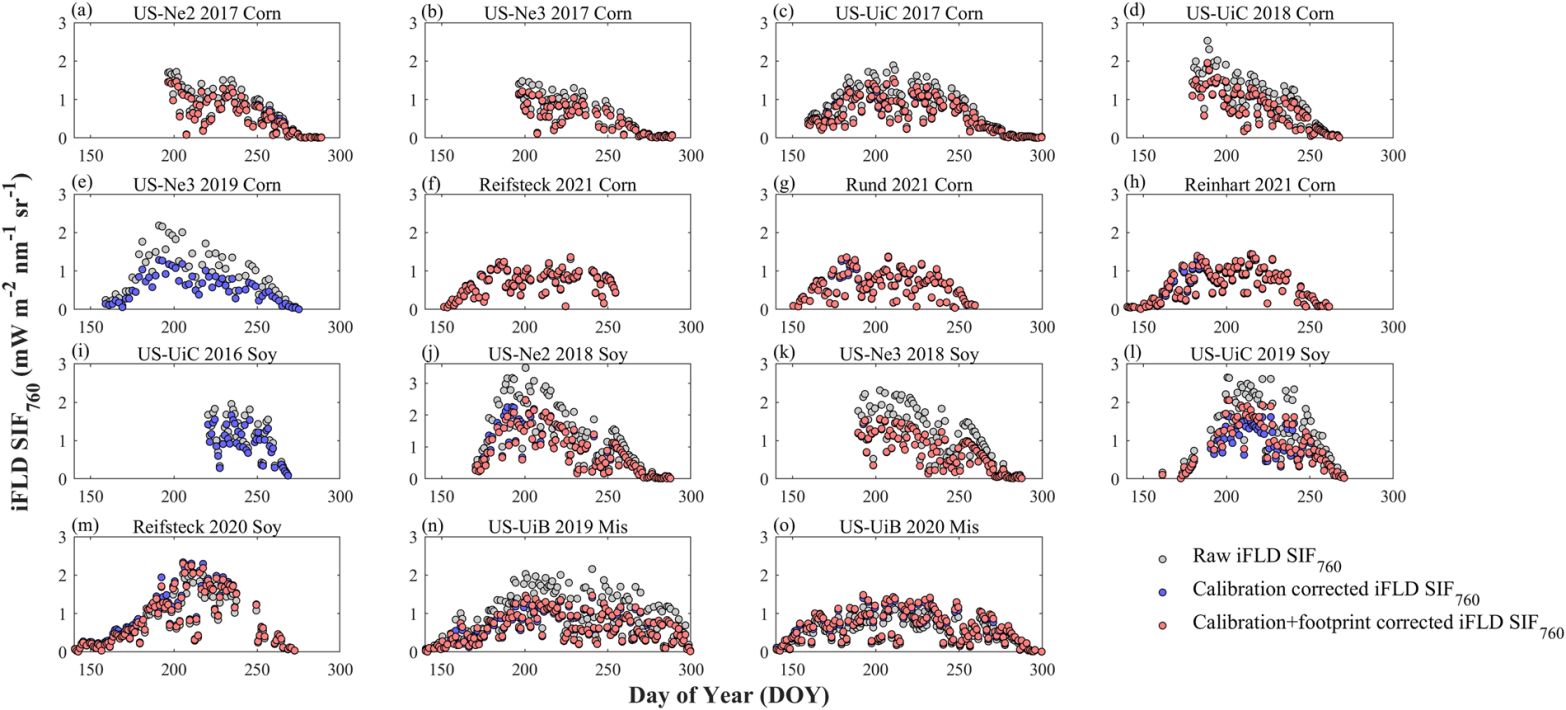
Seasonal variation of daytime average SIF_760_ from local time 8 am to 6 pm at each site-year. Grey, blue, and red circles represent raw iFLD SIF_760_, calibration corrected iFLD SIF_760,_ and calibration + footprint corrected iFLD SIF_760_.

**Fig. 9:**
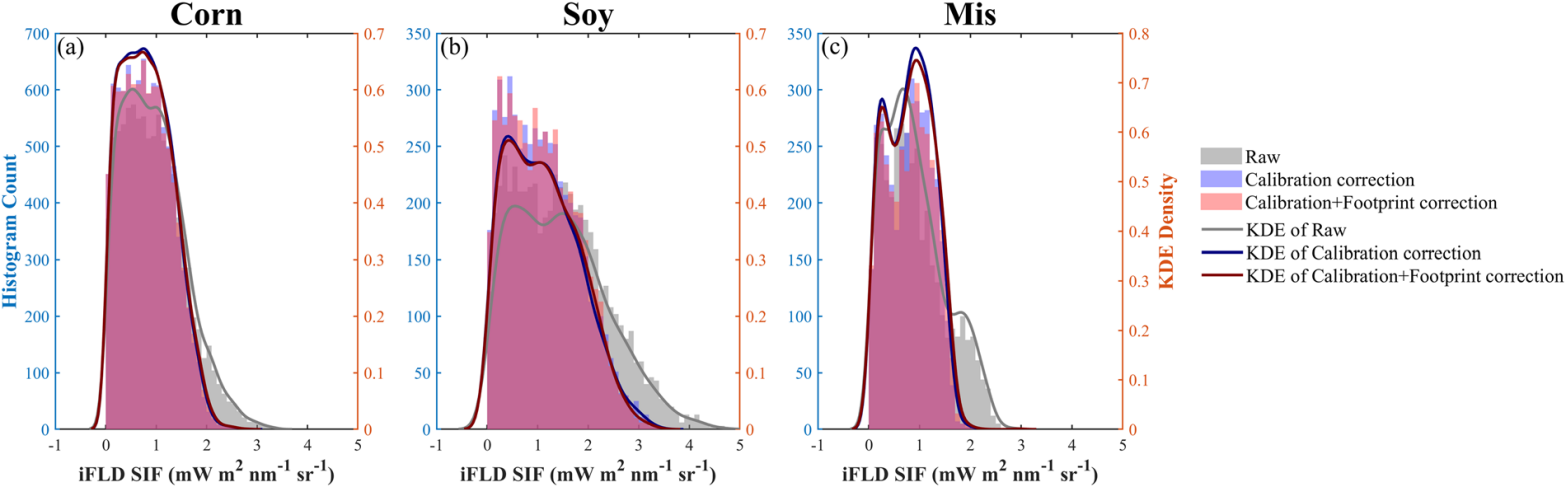
Histogram and Gaussian kernel estimate (KDE) density of peak season half-hourly raw iFLD SIF_760_ (grey), calibration corrected iFLD SIF_760_ (blue) and calibration + footprint corrected SIF_760_ (red) in (**a**) corn, (**b**) soybean, and (**c**) miscanthus.

Radiometric calibration coefficient adjustment decreased the SIF_760_ magnitude for site-years from 2017 to 2019 due to their lower than 1 adjustment factor (Figs. 8, 9). It also decreased the variations of SIF_760_ across different site-years within the same species (Fig. 9). This highlights the importance of this calibration correction since fewer variations of SIF_760_ within the same species are more reasonable when the environmental conditions are similar across different years. Calibration correction did not change the seasonal pattern of SIF_760_ at each site-year because the same adjustment factor was applied to all the data over the season. For all crops, SIF_760_ was near-zero at the start and end of the growing season and increased with the growth of crops and the maximum SIF_760_ were reached at the peak season. Upscaling nadir SIF_760_ to EC footprint had a marginal effect on the magnitude and seasonal pattern of SIF_760_ at all site-years, largely due to the relatively homogeneous field conditions in croplands (Figs. 8, 9). Overall, soybean showed slightly higher SIF_760_ compared to corn and miscanthus, indicated by the higher medium SIF_760_ during the peak growing season shown in Fig. 9. This pattern was consistent among raw SIF_760_, calibration-corrected SIF_760_ and footprint-upscaled SIF_760_. Considering that corn had higher GPP compared to soybean, the slightly lower SIF_760_ combining with higher GPP in corn resulted in different SIF_760_ – GPP relationships between corn and soybean reported in a previous study^66^.

### Variations of VIs in corn, soybean and miscanthus

**Fig. 10:**
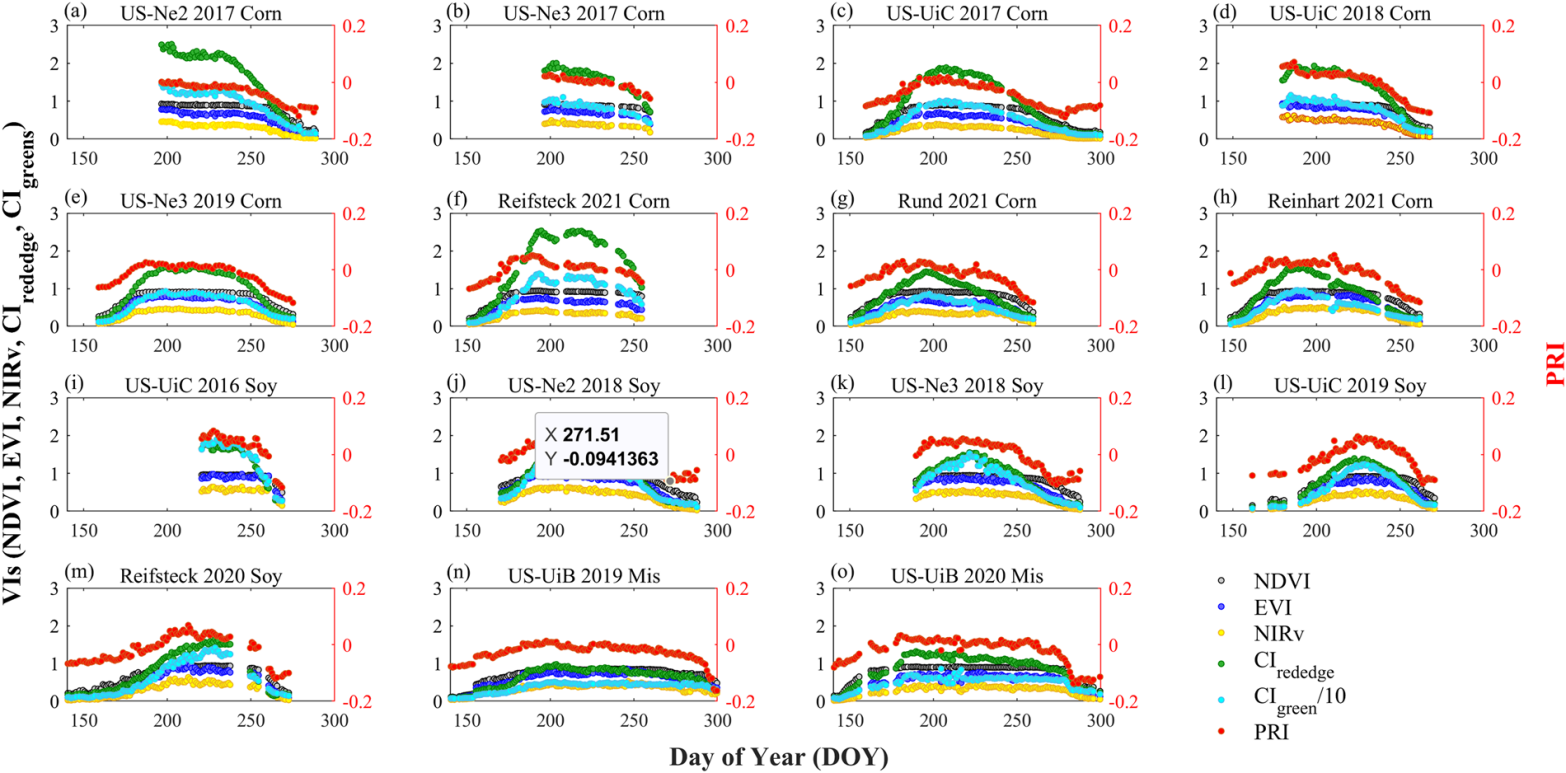
Seasonal variations of daytime average VIs from 8 am to 6 pm at each site-year. Different VIs are represented by different colours, with NDVI by grey circles, EVI by blue circles, NIRv by yellow circles, CI_rededge_ divided by 10 by green circles, CI_green_ divided by 10 by cyan circles, and PRI by red circles. CI_rededge_ and CI_green_ were divided by 10 to match the magnitude of the other VIs.

**Fig. 11:**
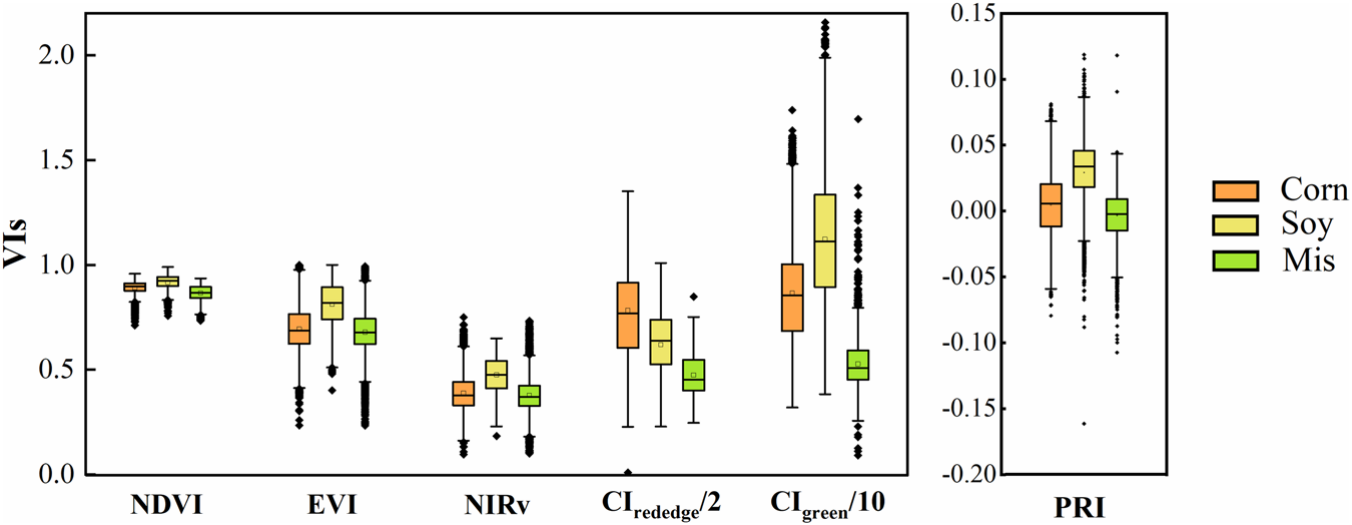
Boxplot of peak season half-hourly NDVI, EVI, NIRv, CI_rededge_ divided by 10, CI_green_ divided by 10, and PRI in corn (orange), soybean (yellow), and miscanthus (green).

The six VIs showed overall similar seasonal patterns at each site-year, with lower values shown at the early and late growing seasons and higher values at the peak season, consistent with the seasonal pattern of SIF_760_ (Fig. 10). NDVI showed a saturated pattern at the peak season. CI_rededege_ and CI_green_ showed similar but larger seasonal variations compared to the other VIs with CI_green_ being noisier than CI_rededge_. Canopy PRI was strongly affected by canopy structure at the seasonal scale; therefore, it showed similar seasonal patterns as EVI and NIRv. Among the three species, soybean showed overall higher peak-season NDVI, NIRv, EVI, and PRI, and corn and miscanthus showed similar magnitudes for these four VIs (Fig. 11). This pattern was consistent with SIF_760_ that higher peak-season SIF_760_ was found in soybean compared to corn and miscanthus. For CI_rededge_, corn showed the highest magnitude followed by soybean and miscanthus. Since CI_rededge_ was calculated with 720–730 nm, the relationship between CI_rededge_ and canopy chlorophyll content was generic for corn and soybean, therefore, higher CI_rededge_ in corn indicated higher canopy chlorophyll content in corn^49^. For CI_green_, the magnitude ranged as soybean > corn > miscanthus. Among the three species, the magnitude of peak-season SIF_760_ was consistent with that of peak-season NDVI, NIRv, and EVI, demonstrating the dominance of the canopy structure on the SIF_760_ signal at crop sites. The overall consistent pattern of SIF_760_ and VIs among corn, soybean and miscanthus indirectly justified the reliability of our retrieved SIF_760_ and VIs^53^.

### Relationships between VIs, APAR and SIF_760_

**Fig. 12:**
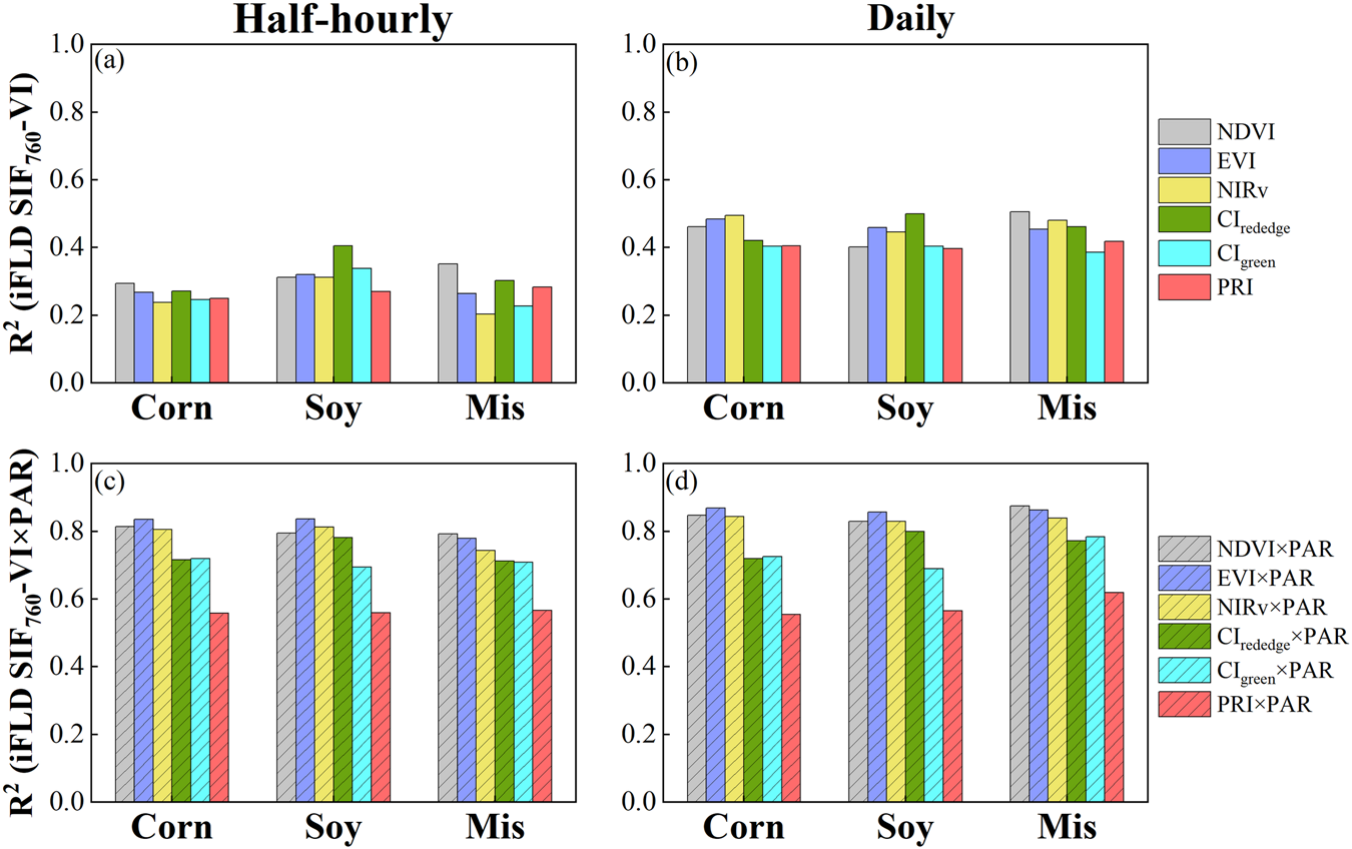
Relationship between calibration corrected iFLD SIF_760_, VI and the product of VI and PAR (VI × PAR) in corn, soybean, and miscanthus. All data available for the same species are combined for this analysis.

Previous studies have demonstrated the dominant role of canopy structure and PAR in interpretating canopy SIF_760_ signal^31,67^. To further validate our SIF_760_ and VIs dataset^53^, we examined the relationship between SIF_760_ and VIs as well as between SIF_760_ and VI × PAR, and decomposed peak-season SIF_760_ into structural, radiation and physiological information. As expected, SIF_760_ and VIs were poorly correlated at the half-hourly scale, with R^2^ ranging from 0.20 to 0.40 across three species and six VIs (Fig. 12). Averaging to the daily scale (daytime average) improved the correlation between SIF_760_ and VIs with R^2^ ranging from 0.39 to 0.51. Incorporating PAR information substantially improved the correlation between SIF_760_ and VIs at both half-hourly and daily scales with R^2^ ranging from 0.56 to 0.88 (Fig. 12). The product of PAR and three structural VIs (NDVI, EVI, and NIRv) showed the highest correlations with SIF_760_, followed by the product of PAR and two chlorophyll indices (CI_rededge_, CI_green_), while the product of PRI and PAR showed the lowest correlation with SIF_760_. This demonstrates the importance of structural information in SIF_760_ at crop sites, as reported in previous studies^31,33^. NIRv did not outperform NDVI and EVI in terms of the correlation with SIF_760_, largely because NDVI and EVI were ratios that were less affected by the calibration process across different site-years while NIRv relied on the near-infrared absolute reflectance which showed larger variations across different site-years. The strong relationship between SIF_760_ and the product of structural VIs and PAR further indirectly supported the credibility of our SIF_760_ and VIs dataset^53^.

**Fig. 13:**
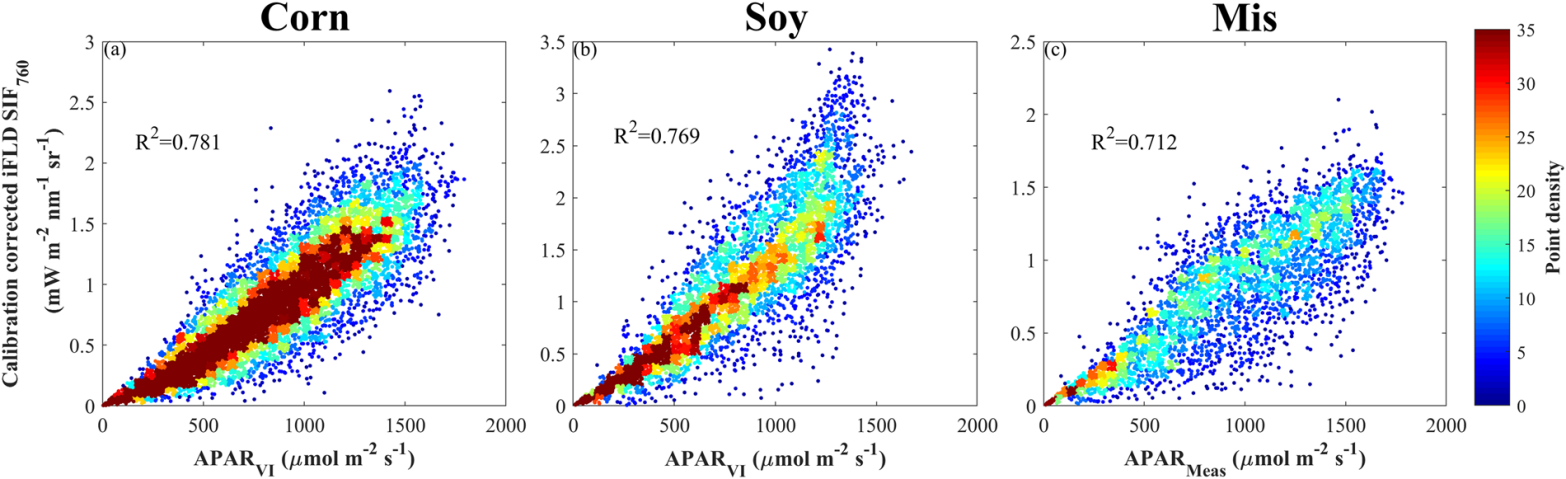
Relationship between peak-season half-hourly APAR and calibration corrected iFLD SIF_760_ in (**a**) corn, (**b**) soybean, and (**c**) miscanthus. APAR is calculated from VI (Rededge NDVI) in corn and soybean (APAR_VI_), while APAR is measured in miscanthus (APAR_Meas_).

**Fig. 14:**
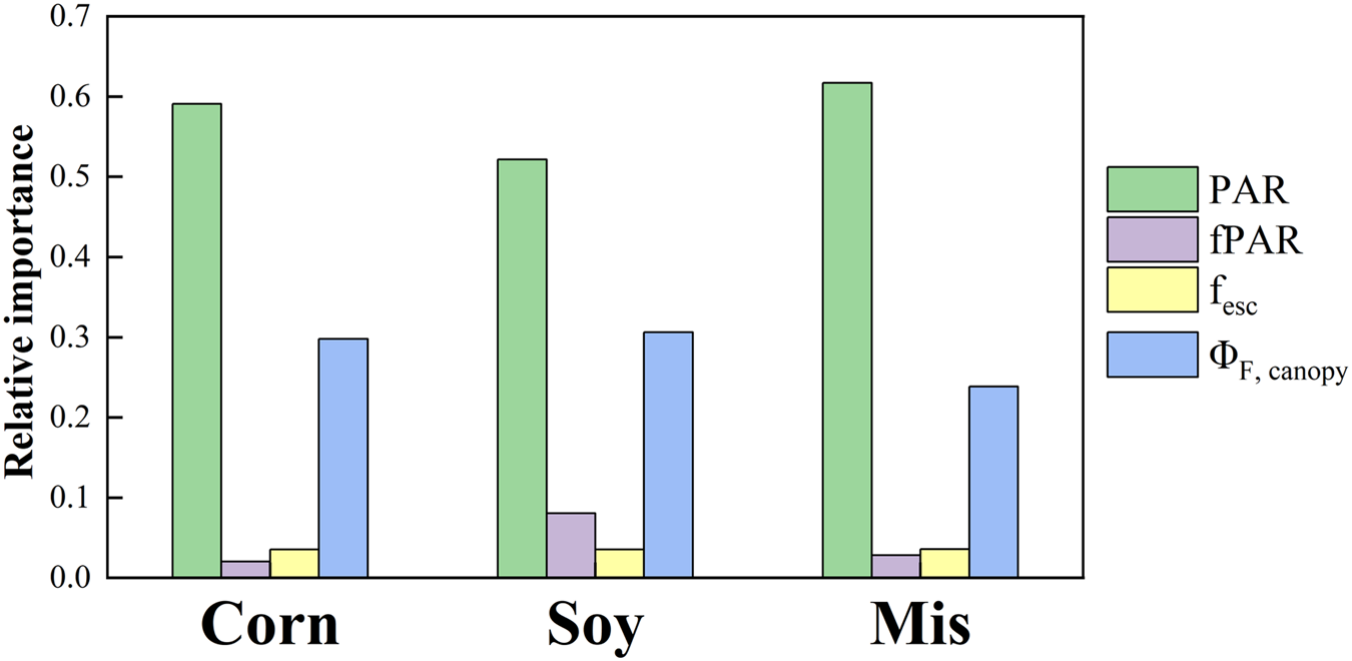
The relative importance of PAR, fPAR, f_esc,_ and Φ_F, canopy_ to peak season calibration-corrected iFLD SIF_760_ for corn, soybean, and miscanthus calculated from the LMG method.

Peak-season half-hourly SIF_760_ was dominated by APAR for all three species(Fig. 13), consistent with the results reported in earlier studies^31,33^. The slightly lower R^2^ in miscanthus was due to the SIF_760_ midday depression under high vapor pressure deficit (VPD), air temperature and PAR conditions^30^. During the peak season when the canopy structure was stable, the contributions of fPAR and f_esc_ to half-hourly SIF_760_ signal were marginal (Fig. 14). PAR and Φ_F, canopy_ explained 52–62% and 24–31% of half-hourly SIF_760_ variations across three species, respectively. This confirmed the contribution of physiological variation to the SIF_760_ signal in cropland, and this physiological component of SIF_760_ is important to capture the early and short-term crop response to stresses^68^. A recent study utilizing part of this SIF_760,_ and VIs dataset has found that Φ_F, canopy_ has the advantage of capturing the physiological responses of crops to water deficit and high temperature over structural proxies such as NIRv^57^.

In conclusion, the collective evidence from all the indirect validation methods employed supports the high quality of our dataset. These validation techniques, encompassing various analytical approaches and comparisons, have collectively corroborated the reliability of the data^53^ we have gathered.

## Usage Notes

To facilitate the effective reuse of our shared far-red SIF and VIs dataset^53^ by other researchers, we provide the following guidelines:

1. We recommend the use of the iFLD-based SIF_760_ retrieval for our dataset. This recommendation is based on our findings that FLD-based SIF_760_ retrieval exhibits lesser sensitivity to enclosure temperature variations compared to the SFM-based retrieval, and the iFLD method demonstrates enhanced sophistication over the sFLD and the 3FLD.
2. Adjusting the radiometric coefficients caused by the degradation of calibrating light source through cross-validation was essential to provide a consistent and less variable SIF_760_ estimate across different site-years.
3. Upscaling ground nadir SIF_760_ to eddy covariance flux footprint may not be necessary in the context of our dataset. This is due to the relatively homogeneous field conditions typical of cropland environments.
4. This dataset could serve as valuable ground validation for satellite products, as well as for modelling related to both radiative transfer and ecosystem dynamics. Additionally, this dataset can be combined with ancillary measurements at leaf and canopy scales to improve the interpretation and understanding of the SIF signal as well as the relationship between SIF and photosynthesis.

## Data Availability

The code implementation was done in Matlab (2017a). The functions for far-red SIF and VIs estimation used in this study are available at https://github.com/wugh16/SIF_VI_process_functions.git. The far-red SIF and VIs data is available at 10.3334/ORNLDAAC/2136^53^.
